# *Hippophae rhamnoides* as novel phytogenic feed additive for broiler chickens at high altitude cold desert

**DOI:** 10.1038/s41598-018-24409-9

**Published:** 2018-04-13

**Authors:** Sahil Kalia, Vijay K. Bharti, Arup Giri, Bhuvnesh Kumar, Achin Arora, S. S. Balaje

**Affiliations:** 1 Defence Institute of Physiology and Allied Sciences (DIPAS), New Delhi, India; 2 Defence Institute of High Altitude Research (DIHAR), DRDO, C/o- 56 APO, Leh-Ladakh, (J and K) India

## Abstract

Extremes of climate and hypobaric hypoxia cause poor growth performance in broiler chickens at high altitude. The present study examined the potential of *Hippophae rhamnoides* extract as phytogenic feed additive for broilers reared at 3500 m above mean sea level (MSL). Higher content of phytomolecules were recorded during characterization of the extract. Immunomodulatory activity of extract was observed in chicken lymphocytes through *in-vitro* studies. Thereafter, for *in vivo* study, 105 day old Rhode Island Red (RIR) Cross-bred chicks were randomly distributed in to control and treatments T1, T2, T3, T4, T5, and T6 which were supplemented with *H. rhamnoides* aqueous extract along with basal diet, at level of 100, 150, 200, 300, 400, and 800 mg/kg body weight of chicken, respectively. Among the experimental groups, birds in the T3 group represent the highest body weight. Furthermore, treatment group birds had shown better physio-biochemical indices as compared to control group birds. Interestingly, lower mortality rate due to ascites and coccidiosis was recorded in treatment groups and therefore, higher net return was observed. Hence, present investigation demonstrated the beneficial effect of *H. rhamnoides* extract (@200 mg/kg) at high altitude and therefore, may be used in formulation of feed additive for poultry ration.

## Introduction

There has been significant improvement in the growth performance in broiler poultry industry has been achieved in India over the last twenty years. But in the stressful environmental conditions of high altitude cold desert Himalayas which are depicted with supreme temperature differences (from +35 °C to −35 °C), hypobaric hypoxia, excessive Ultraviolet radiations, very less availability of water vapours in the atmosphere and shortage of animal food, the growth rate of poultry chickens is severely affected and due to which poultry farming is not more successful in this part of world^1–3^. These unfavourable environmental conditions cause variation in physiological functions which could results in metabolic disorders and deficiency in essential nutrients^4^. Low partial pressure of oxygen (PO_2_) at high altitude alters the electron transport mechanisms of mitochondria which leads to the excessive formation of free radicals causing oxidative stress which results in the more oxidative damage to macromolecules and impaired immune status in livestock animals^2,3^. Free radicals are highly unstable, very reactive radicals which can start chain reaction once formed and are capable of damaging molecules such as DNA, proteins, lipids or carbohydrates. The damage to biological molecules ultimately compromises growth, development, immunocompetence and reproduction in livestock^5,6^. However, the Himalayan region is abundant in several plants species which are widely used in traditional systems of medicines particularly *‘Amchi’* system of medicine for treatment of various ailments^7^. *‘Amchi’* system of medicine which is also called ‘Sowa Rigpa’ (Science of healing) is one of the oldest, living and well documented medical tradition of the world which has been popularly practice in Tibet, Magnolia, Bhutan, some parts of China, Nepal, and trans-Himalayan regions of India^8,9^. Incorporation of phytomolecules rich herbal plant extract in poultry diet in the form of feed additives would have been promising benefits on health and nutritional status of the chickens.

*Hippophae rhamnoides* commonly known as seabuckthorn (SBT) is a temperate shrub growing at an altitude of 3000 to 4500 m above MSL in high altitude Himalayas^10^. Every part of the *H. rhamnoides* plant is an abundant source of bioactive plant phytomolecules such as polyphenols, flavonoids, vitamins, carotenoids, organic acid, polyunsaturated fatty acids, and amino acids^10,11^. In the traditional ayurvadic system of medicine the extract of *H. rhamnoides* fruits has been used as an immunomodulator and anti-stress agent^12^. Previous studies reported various pharmacological activities of *H. rhamnoides* including antioxidant^13^, immunomodulatory^14^, anti-stress^15^, anti-tumor^16^, hepato protective^13^, and radio protective^17^. These medicinal effects of *H. rhamnoides* have been attributed to presence of high antioxidant content in this plant^11,18^. It has been suggested by Biswas *et al*.^19^ that *H. rhamnoides* seeds, leaves, and fruit residues are ideal source of feeding material for the poultry chickens in high altitude region of trans-Himalayan. Ma *et al*.^20^ reported an significant enhancement in broilers performance after supplementation of flavonoids of *H. rhamnoides* fruits in broilers diet. However, still so far, no previous study has ever evaluates the feeding potential of *H. rhamnoides* in broiler chicken diet at high altitude. Therefore, the current study was performed to determine the dietary supplemental effect of *H. rhamnoides* extract on broiler growth performance, survivability rate, physio-biochemical indices, and cost economics of their rearing at high altitude and evaluate its potential as a phytogenic feed additive.

## Methods

### Plant material and extraction

Fresh *H. rhamnoides* fruits were gathered from the market of Leh district through local vendors, and thereafter, washed upon arrival at the laboratory and then shed dried at room temperature for 30 days. After that, dried fruit samples were powdered and extracted with distilled water in a soxhlet apparatus for 24–48 hrs.

Extract yield was calculated on the basis of the following equation:

$${\rm{Extract}}\,{\rm{Yield}}\,( \% )={\rm{Total}}\,{\rm{amount}}\,{\rm{of}}\,{\rm{extract}}/{\rm{Total}}\,{\rm{amount}}\,{\rm{of}}\,{\rm{powder}}\,{\rm{sample}}\ast 100.$$

### Characterization of the extract

*H. rhamnoides* fruit extract was characterized for total polyphenols, flavonoids, and carotenoids contents, and also analysed for free radical scavenging activity and total antioxidant capacity (TAC).

### TAC

TAC in extract sample was determined by ferric reducing antioxidant power (FRAP) assay as suggested by Benzie and Strain^21^. Results were expressed as µM Fe (II)/g of extract.

### Free radical scavenging capacity

The effect of *H. rhamnoides* fruit extract on scavenging of 2,2-Diphenyl-1-picrylhydrazyl (DPPH) radical was determined using the method proposed by Brand-William *et al*.^22^. For this assay, 0.1 mM solution of DPPH in methanol was prepared and 600 µL of this solution was allowed to react with 30 µL of sample (extract). A control was treated with 30 µL of solvent instead of the sample. Solution was centrifuged (Heal Force ®, Neofuge 23 R, Shanghai, China) after incubation of 30 min at room temperature and supernatant was transferred in to 96 well microtiter plate and the absorbance was recorded at 517 nm using of ELISA microplate reader (Spectromax M2e, Molecular Devices, California, USA). Ascorbic acid was used as reference standard. Scavenging of DPPH radical by the sample (fruit extract and plasma) was calculated by the following equation:

$${\rm{DPPH}}\,{\rm{radical}}\,{\rm{scavenging}}\,{\rm{activity}}\,( \% )=[({{\rm{Ab}}}_{{\rm{control}}}\,-\,{{\rm{Ab}}}_{{\rm{sample}}})]\,/({{\rm{Ab}}}_{{\rm{control}}})]\times 100$$

Where Ab _control_ is the absorbance of control, and Ab _sample_ is the absorbance of sample or standard.

### 2,2-azino-bis(3-ethylbenzothiazoline-6-sulphonic acid) ABTS radical scavenging capacity

The potential of *H. rhamnoides* extract to scavenge ABTS radical was determined by the method proposed by Re *et al*.^23^. The stock solution for this assay comprised of 7 mM ABTS solution and 2.4 mM potassium persulphate solution. Two stock solutions were mixed in equal quantities for the preparation of working solution followed by incubation of 12 h in dark at room temperature. After incubation, 1 ml of working solution was mixed with 60 ml of 96% ethanol for obtaining an initial absorbance of 0.700 ± 0.02 at 734 nm. Fruit extract (33.30 µL) was allowed to react with ABTS^+^ solution (266.70 µL) and the decrease in the absorbance was measured after 7 min at 734 nm. Ascorbic acid was used as reference standard. The ABTS^+^ scavenging capacity of fruit extract was calculated as follows:

$${\rm{ABTS}}\,{\rm{radical}}\,{\rm{scavenging}}\,{\rm{capacity}}\,( \% )=[({{\rm{Absorbance}}}_{{\rm{control}}}-{{\rm{Absorbance}}}_{{\rm{sample}}})]\,/({{\rm{Absorbance}}}_{{\rm{control}}})]\times 100.$$

Where Absorbance _control_ is the absorbance of control, and Absorbance _sample_ is the absorbance of sample or standard.

### Total phenolic content

Total phenolic content in *H. rhamnoides* fruit extract was evaluated by Folin-Ciocalteu calorimetric method as suggested by Gao *et al*.^24^.

### Total flavonoid content

Total flavonoid content in *Hippophae rhamnoides* fruit extract was estimated by the method as suggested by Ordonez *et al*.^25^. For this assay, 0.5 mL of extract was allowed to mix with 0.5 mL of 2% aluminium chloride (AlCl_3_) ethanolic solution. After 1 hr of incubation at room temperature the absorbance was measured at 420 nm. Quercetin was used as standard and total flavonoid content was indicated as mg of quercetin equivalent.

### Determination of carotenoids

These were estimated by the method as suggested by Ranjith *et al*.^26^. For this assay, 0.5 mL of 5% sodium chloride (NaCl) and 2 mL of hexane was mixed with fruit extract. After that, the solution was vortexed for 30 seconds followed by centrifugation for 10 minutes. Absorbance was measured at 460 nm. *β*-carotene was used as reference standard and total carotenoids in fruit extract were indicated as mg of *β*-carotene equivalent.

### *In vitro* evaluation for dose efficacy of *H. rhamnoides* extract

To determine the efficacy of *H. rhamnoides* fruit extract, it’s antioxidative and cytoprotective activities were initially assessed in chicken peripheral blood lymphocytes (PBL) prior to *in vivo* studies.

### Blood sampling & separation of peripheral blood mononuclear cells (PBMC)

We took 3 ml of blood samples from wing vein of chickens and collected those samples into sterile plastic tubes containing ethylenediaminetetraacetic acid (EDTA) as an anticoagulant. For separation of PBMC, whole blood was first diluted with phosphate buffered saline (PBS, Ca^2+^ and Mg^2+^ free, Himedia) in 1:1 ratio and thereafter smoothly over layered on Histopaque-1077 (Sigma-Aldrich, St. Louis, MO) in falcon tube and centrifuged for 30 m at 400 × g. We recovered the PMBC from gradient interface, washed them twofold with Ca^2+^ and Mg^2+^ free PBS, and centrifuged at 200 × g for 10 m. The last washing was performed with RPMI-1640 medium (R1145, Sigma-Aldrich). The pellets were then resuspended in 10% fetal bovine serum (FBS) rich RPMI-1640 medium. Plastic adherence technique proposed by Gupta *et al*.^27^ was used to separate a non-adherent (lymphocytes) cells from the adherent (monocytes) one.

### Cell Culture

PBL suspension (100 µL/well) was cultured in microtiter plate with 100 µL/well of different dose concentrations of *H. rhamnoides* extract (100, 200, 400, 800 ng/mL, and 1, 2, 4, 8, 50, 100, 200, 400 μg/mL), 1 µg/mL of concanavalin A as positive control, and medium as negative control, at 41 °C in a 5% CO_2_ incubator for 24 h.

### 3-(4,5-dimethylthiazol-2-yl)-2,5-diphenyltetrazolium bromide (MTT) proliferation assay

The calorimetric method of MTT assay described by Mosmann^28^ was used to assess the proliferative activity of extract in chicken PBL. Following incubation of chicken PBL with *H. rhamnoides* extract for 24 h, 50 µL of MTT solution was added to each well and after 4 h of incubation, 100 µL of dimethyl sulfoxide (DMSO) was added to solubilize the formazan product. By using microplate reader absorbance was taken at 570 nm.

$$ \% \,{\rm{Cell}}\,{\rm{viability}}={\rm{Absorbance}}\,{\rm{of}}\,{\rm{Test}}/{\rm{Absorbance}}\,{\rm{of}}\,{\rm{Control}}\times 100.$$

### Cytoprotective assay against hydrogen peroxide (H_2_O_2_) induced toxicity

To analyse the cytoprotective activity of plant extract against the toxic effect of H_2_O_2_, PBL cells were first cultured into 96 well plates and incubated for 24 h at 41 °C. Thereafter, cells were simultaneously treated with different concentrations of *H. rhamnoides* extract and 100 µm H_2_O_2_ (Merck, India) for 2 h. MTT assay was used to determine the cell viability.

### *In vivo* experiment

Institutional Animal Ethics Committee of DIHAR approved the field experiment and all the methods were performed as per the guidelines of animal experimentation. The experiment was carried out under deep litter system in the solar poultry house of DIHAR having a stacking density of 0.80 square feet per bird in 2 × 2 feet of pen size (5 birds/pen). The ambient temperature of the house was maintained at 25–32 °C by using local bukhari (a heating device). A total of 105 one day old RIR cross-bred broiler chickens were randomly distributed to seven experimental groups as per completely randomized design. There were 3 replications per treatment with 5 chickens per replicate pen. Chickens in the control group were fed the basal diet whereas chickens in the six treatment groups were supplemented with aqueous extract of *H. rhamnoides* fruit in drinking water @ 100 mg/kg body weight of chicken (T1), @ 150 mg/kg body weight of chicken (T2), @ 200 mg/kg body weight of chicken (T3), @ 300 mg/kg body weight of chicken (T4), @ 400 mg/kg body weight of chicken (T5), and @ 800 mg/kg body weight of chicken (T6), respectively, in addition of basal diet. Experiment was conducted from 0 to 42 days of broiler chick age. A standard in house feed formula specific for high altitude poultry chickens developed by our laboratory was used to formulate the basal diet^2^. All the birds were given the same basal diet, included a starter diet (21.56% protein, 12.97 MJ/Kg ME, 1.02% calcium, and 0.48% phosphorus) from day 1 to day 21 and a finisher diet (19.31% protein, 13.38 MJ/Kg ME, 0.94% calcium, and 0.42% phosphorus) from day 22 to d 42. The ingredients and analysed composition of basal diet are presented in Table 1. On 7^th^ day, all the chickens were vaccinated for Newcastle disease. Every chicken was individually weighed at each week interval. Throughout the experiment, water and feed intake was measured. During the experimental trial autopsy inspection of dead birds was done to find out the cause of death. Economics of the experiment was also estimated based on the rearing cost of chickens.

**Table 1:** Ingredients and chemical composition of basal diet.

Ingredients (% diet)	Starter diet (0–21 day)	Finisher diet (22–42 day)
Maize	59.00	58.00
Soyabean (Solvent extracted)	33.18	21.12
Soyabean (Full fat)	—	9.58
Soyabean oil	2.00	2.55
Fish Meal	2.15	—
Wheat bran	—	5.08
Salt (Nacl)	0.15	0.15
Limestone	1.50	1.50
Dicalcium phosphate	1.50	1.50
Lysine	0.13	0.13
Methionine	0.19	0.19
Vitamin & Mineral premix*	0.20	0.20
Total	100	100
Calculated composition		
Protein (%)	21.56	19.31
ME (MJ/Kg)	12.97	13.38
Calcium (%)	1.02	0.94
Phosphorus (%)	0.48	0.42

*Vitamin and mineral premix supplied per kilogram of diet: 14000 IU of vitamin A, 70 mg of vitamin E, 3000 IU of vitamin D_3,_ 4 mg of vitamin K, 3 mg of thiamine, 10 mg of vitaminB_2_, 8 mg of vitamin B_6_, 0.04 mg of vitamin B_12,_ 48 mg of niacin, 20 mg of calcium d-pantothenate, 500 mg of choline chloride, 0.20 mg of biotin, 1.8 mg of folic acid, 80 mg of manganese, 70 mg of zinc, 50 mg of iron, 10 mg of copper, 3 mg of iodine, 0.4 mg of selenium, and 0.2 mg of cobalt.

### Blood collection

For collection of blood sample we randomly picked nine chickens from each group (3 chickens from each replicate pen) at 0, 21, and 42 day and took 3 ml of blood samples from wing vein of chickens and collected those samples into sterile plastic tubes containing EDTA as an anticoagulant. EDTA tubes containing blood were centrifuged at 3500 RPM for 10 m at 4 °C to obtain clear plasma and stored at −80 °C until use.

## Physio-biochemical indices

### Determination of plasma antioxidant parameters

Plasma TAC and free radical scavenging activity were analysed as described earlier in the section. For plasma TAC the results were indicated as FRAP value (µM Fe (II)/L of plasma).

### Lipid peroxidation assay (LPO)

Lipid peroxidation assay was performed by measuring malondialdehyde (MDA) concentration in plasma samples according to the previously described method^29^. For this assay, 375 mg of thiobarbituric acid (TBA) was dissolved in 2 mL of 0.25 N hydrochloric acid (HCL) followed by 15 g of trichloroacetic acid (TCA) for a final volume of 100 mL. To properly dissolve TBA, solution was heated in water bath (GFL water bath, Burgwedal, Germany) at 55 °C for 15 minutes. Thereafter, 500 µL of TCA-TBA-HCL solution was mixed properly with 250 µL of plasma sample. This solution was again heated in boiling water bath for 15 minutes. After cooling, to remove flocculent precipitate solution was centrifuged and absorbance was taken at 535 nm against a blank that contained all reagents expects the plasma sample.

### Determination of blood biochemical parameters

The level of total protein, albumin, glucose, creatinine, alanine transaminase (ALT), aspartate transaminase (AST), low density lipoprotein (LDL), triglyceride, cholesterol, and high density lipoprotein (HDL) in chicken plasma samples were evaluated with commercial biochemical kits (Span Diagnostics, India) according to suggested methodology.

### Statistical analysis

Data were analyzed by one way analysis of variance (ANOVA) using completely randomized design. Values were expressed as mean ± standard error. Statistical significant values were assumed at *P* < 0.05. For growth performance, 3 replicates pen per treatment (5 broiler chickens per replicate pen) served as experimental unit.

## Results

### Free radical scavenging capacity of extract

The details of free radical scavenging of aqueous extract of *H. rhamnoides* compared to positive control ascorbic acid are shown in Table 2. *H. rhamnoides* scavenged the DPPH and ABTS radical at concentration of 20 to 100 µg/ml and scavenging activity increases with increase in the extract concentration. Positive control ascorbic acid was also found to produce inhibition of free radicals at similar concentration.

**Table 2:** Free radical scavenging activity of extract.

Inhibition (%)				
DPPH radical scavenging capacity		ABTS radical scavenging capacity		
Concentration (µg/ml)	*H. rhamnoides* extract	Ascorbic acid	*H. rhamnoides* extract	Ascorbic acid
20	31.85 ± 0.65	39.57 ± 0.76	15.63 ± 0.40	21.36 ± 1.12
40	32.01 ± 0.78	45.40 ± 0.89	19.27 ± 0.25	29.37 ± 0.45
60	35.89 ± 0.81	49.80 ± 0.63	24.41 ± 0.39	35.86 ± 0.54
80	38.25 ± 0.67	53.98 ± 0.57	30.40 ± 0.63	41.18 ± 0.71
100	39.47 ± 0.90	60.59 ± 1.08	35.25 ± 0.84	55.94 ± 0.96

Scavenging capacity of aqueous extract of *H. rhamnoides* was determined against DPPH and ABTS radical. Ascorbic acid was used as a reference standard. Values are given as mean ± S.E.

### TAC

TAC of *H. rhamnoides* was determined by FRAP assay and was recorded to be 425.54 ± 16.14 µM Fe (II)/g of extract (data not shown).

### Phytomolecules content

Total polyphenolic content in *H. rhamnoides* extract was recorded to be 76.28 ± 3.25 mg gallic acid (GAE)/g of extract. Flavonoid content was recorded to be 35.14 ± 2.18 mg quercetin (QE)/g of extract and carotenoid content was recorded to be 4.19 ± 0.70 mg/100 g of extract (data not shown).

### *In vitro* efficacy

**Figure 1:**
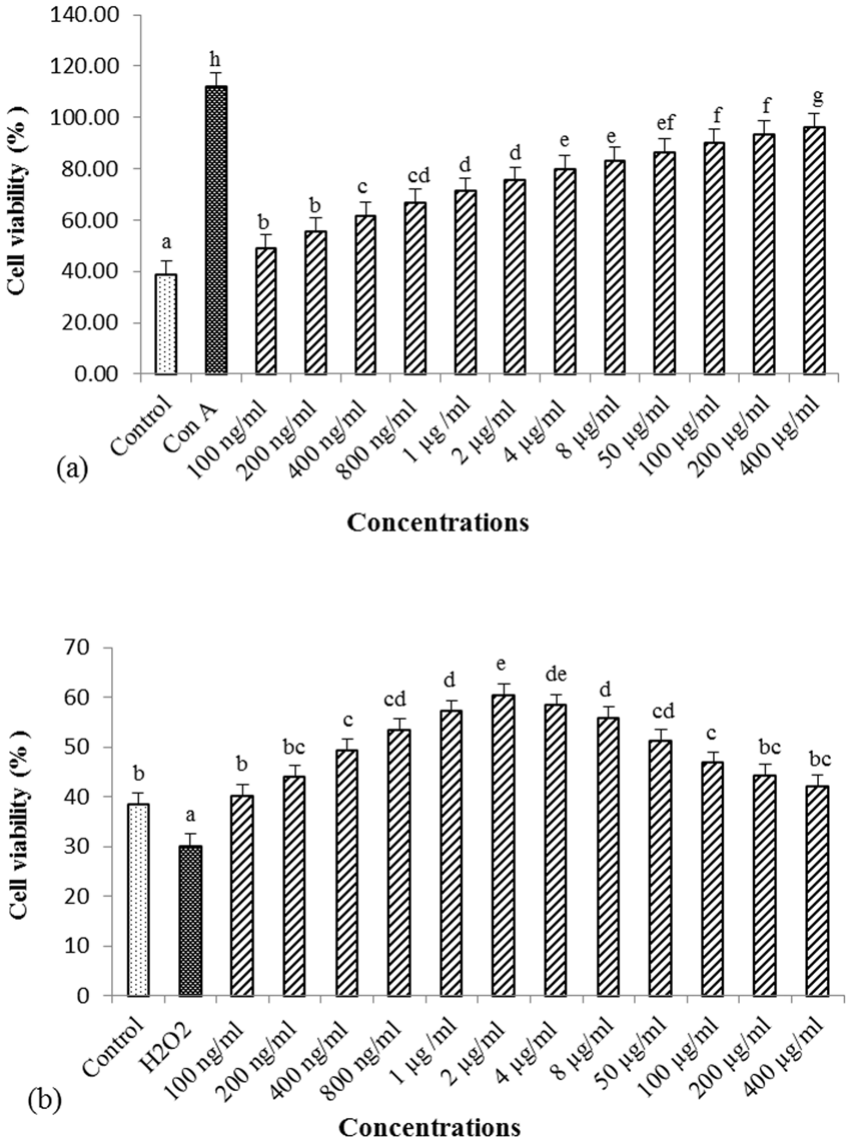
*In vitro* efficacy of *H. rhamnoides* extract. (**a**) Effect of aqueous extract of *H. rhamnoides* on chicken PBL proliferation. Cells were treated with different concentrations of extract (100 ng/mL-400 µg/mL) and concanavalin A (positive control) for 24 hrs. Each value was compared with untreated control cells as well as with in different dose concentrations. (**b**) Cytoprotective activity of extract against H_2_O_2_ induced toxicity in chicken PBL. Cells were incubated simultaneously with different concentrations of extract (100 ng/mL-400 µg/mL) and H_2_O_2_ for 2 hrs. Each value was compared with H_2_O_2_ stimulated cells as well as with in different dose concentrations. Bars having different superscript (^a, b, c, d, e, f, g^) differ significantly (*P* < *0.05*).

Treatment of *H. rhamnoides* extract with chicken lymphocytes increase the proliferation of cells at all dose concentrations in between 100 ng/mL to 400 µg/mL as compared with the untreated control cells (Fig. 1a). However, proliferation of *H. rhamnoides* stimulated PBL was less as compared to proliferation of Concanavalin A stimulated PBL. *H. rhamnoides* extract was also found to reduce the H_2_O_2_ induced oxidative stress in lymphocytes at similar concentration as compared with H_2_O_2_ stimulated control cells (Fig. 1b). The highest cytoprotective activity of *H. rhamnoides* was recorded at 2 µg/ml dose concentration.

### Growth performance

Chickens in T3 group had significantly higher body weight as compared with control and other treatment groups at 21 days (Table 3). Whereas, no differences were observed in body weight among the control and other treatment groups at 21 days. Further, at 42 day, we observed a significantly higher body weight in all treatment groups as compared with the control group and within treatment groups, birds in the T3 group represented the highest body weight. Throughout the experiment cumulative feed and water intake did not differ among the experimental groups. Feed conversion ratio (FCR) value in the T3 group was found to be significantly improved among the experimental groups (Table 3).

**Table 3:** Effect of aqueous extract of *H. rhamnoides* on growth performance of broiler chickens.

Treatments							
Parameters	Control	T1	T2	T3	T4	T5	T6
Initial average body weight (gm/chick)	38.86 ± 0.60	39.60 ± 0.58	39.93 ± 0.57	38.20 ± 0.52	38.53 ± 0.58	38.20 ± 0.51	39.33 ± 0.54
Average weight at 21 day (gm/chick)	192.80^a^ ± 3.50	194.26^a^ ± 1.97	192.46^a^ ± 3.45	222.13^b^ ± 5.26	190.26^a^ ± 3.82	189.20^a^ ± 5.99	191.06^a^ ± 8.41
Average weight at 42 day (gm/chick)	356.75^a^ ± 10.06	410.70^b^ ± 10.38	402.53^b^ ± 12.32	470.33^c^ ± 12.68	394.26^b^ ± 6.69	409.20^b^ ± 6.78	392.14^b^ ± 5.55
Cumulative feed intake up to 42 day (gm/chick)	1519.20 ± 9.65	1521.08 ± 9.54	1520.37 ± 8.70	1530.27 ± 14.59	1544.58 ± 16.48	1518.62 ± 11.63	1527.50 ± 11.39
Feed conversion ratio at 42 day	4.78^d^ ± 0.08	4.10^b^ ± 0.06	4.19^b,c^ ± 0.02	3.54^a^ ± 0.08	4.34^c^ ± 0.06	4.09^b^ ± 0.05	4.33^c^ ± 0.04
Cumulative water intake up to 42 day (ml/chick)	2138.76 ± 17.37	2140.71 ± 11.67	2145.69 ± 12.92	2140.39 ± 11.39	2155.02 ± 16.36	2150.71 ± 11.86	2155.43 ± 11.96

Chickens in the control group were fed the basal diet whereas the six treatment groups, in addition of basal diet received aqueous extract of *H. rhamnoides* in drinking water @ 100 mg/kg body weight of chicken (T1), @ 150 mg/kg body weight of chicken (T2), @ 200 mg/kg body weight of chicken (T3), @ 300 mg/kg body weight of chicken (T4), @ 400 mg/kg body weight of chicken (T5), and @ 800 mg/kg body weight of chicken (T6), respectively from days 0 to 42. Results are presented as mean ± S.E. Experimental unit 3 replicates pen (5 broiler chickens per replicate pen). Means bearing the different superscripts ^(a, b, c, d)^ in a row differ significantly (*P* < 0.05).

### Mortality rate and economics

Maximum mortality rate (26.67%, 4/15) was observed in the control group (Table 4) chicken followed by T1, T2, T4, T5 and T6 (13.30%, 2/15), which was followed by T3 (6.67%, 1/15). Autopsy inspection showed 13.30%, 6.67%, 6.67%, 0%, 0%, 0%, and 6.67% mortality in chickens caused by ascites and 6.67%, 0%, 6.67%, 0%, 0%, 6.67%, and 0% mortality caused by coccidiosis in control, T1, T2, T3, T4, T5, and T6 groups, respectively.

**Table 4:** Economics and mortality rate (%) in chickens supplemented with *H. rhamnoides* extract.

Description	Control	T1	T2	T3	T4	T5	T6
Total mortality (%)	26.67	13.30	13.30	6.67	13.30	13.30	13.30
Mortality by ascites (%)	13.30	6.67	6.67	0.00	0.00	0.00	6.67
Mortality by coccidiosis (%)	6.67	0.00	6.67	0.00	0.00	6.67	0.00
Mortality by other reasons (%)	6.67	6.67	0.00	6.67	13.33	6.67	6.67
Cost of extract/chicken (Rs.)	Nil	0.69	1.02	1.58	1.96	2.74	5.40
Cost of feed/chicken (@25/Kg Rs.)	37.98	38.02	38.00	38.26	38.61	37.97	38.18
Total feed cost/bird (Rs.)	37.98	38.71	39.02	39.84	40.57	40.71	43.58
Sale of chicken at 42 day (@Rs. 200/Kg live weight)*	71.35	82.14	80.50	94.06	78.85	81.84	78.43
Loss due to mortality (Rs.)^**†**^	285.40	164.28	161.00	94.06	157.70	163.69	156.87
Total benefit per group (Rs.)^**‡**^	—	121.12	124.40	191.34	127.70	121.70	128.53

*Due to limited availability of fresh chickens at high altitude the rates are very high. ^**†**^Loss due to mortality = Sale cost per chicken × total mortality. ^**‡**^Total benefit per group = Loss from mortality in control – loss from mortality in treatment.

We also estimated the economy of the experiment based on the rearing cost of chickens in each group. Extra price of the extract was added to feed price whereas other expenses stayed unchangeable. *H. rhamnoides* decreased the mortality rate in chickens and which subsequently higher net return (Table 4).

### Plasma blood biochemical status in chickens

The supplementation of *H. rhamnoides* extract significantly increased the concentration of total protein, albumin, and globulin in treatment group birds (Table 5). Moreover, birds in the T3 group represent the highest level of total protein and globulin at 42 day of age. A significant decrease in albumin to globulin (A/G) ratio was noticed in treatment group birds at both 21 and 42 days of age. Within the treatment groups, significantly lowest A/G ratio was noticed in T3 group birds at 42 day of age.

**Table 5:** Effect of *H. rhamnoides* extract on total protein, albumin, globulin, and A/G ratio in broiler chickens.

**Groups**	0 day	21st day	42nd day
**Total protein (g/dL)**			
Control	3.36 ± 0.18	3.51^a^ ± 0.07	3.57^a^ ± 0.13
T1	3.31 ± 0.15	4.72^b^ ± 0.09	5.64^c^ ± 0.08
T2	3.35 ± 0.13	4.76^b^ ± 0.14	5.23^b^ ± 0.09
T3	3.33 ± 0.13	4.89^b^ ± 0.12	5.77^d^ ± 0.11
T4	3.40 ± 0.14	4.65^b^ ± 0.13	5.30^b^ ± 0.27
T5	3.37 ± 0.17	4.74^b^ ± 0.10	5.12^b^ ± 0.21
T6	3.34 ± 0.14	4.60^b^ ± 0.14	5.51^c^ ± 0.13
**Albumin (g/dL**)			
Control	2.08 ± 0.12	2.18^a^ ± 0.08	2.23^a^ ± 0.15
T1	2.05 ± 0.12	2.88^b^ ± 0.10	3.10^b^ ± 0.08
T2	2.04 ± 0.11	2.87^b^ ± 0.11	2.98^b^ ± 0.06
T3	2.03 ± 0.16	2.91^b^ ± 0.21	3.05^b^ ± 0.19
T4	2.06 ± 0.15	2.78^b^ ± 0.17	3.02^b^ ± 0.06
T5	2.07 ± 0.12	2.80^b^ ± 0.11	2.89^b^ ± 0.08
T6	2.06 ± 0.13	2.79^b^ ± 0.15	3.00^b^ ± 0.14
**Globulin (g/dL)**			
Control	1.28 ± 0.08	1.33^a^ ± 0.15	1.34^a^ ± 0.20
T1	1.26 ± 0.10	1.84^b^ ± 0.18	2.54^b^ ± 0.19
T2	1.31 ± 0.14	1.89^b^ ± 0.16	2.25^b^ ± 0.13
T3	1.30 ± 0.19	1.98^b^ ± 0.23	2.72^c^ ± 0.28
T4	1.34 ± 0.11	1.87^b^ ± 0.21	2.28^b^ ± 0.23
T5	1.30 ± 0.09	1.94^b^ ± 0.15	2.23^b^ ± 0.23
T6	1.28 ± 0.09	1.81^b^ ± 0.18	2.51^b^ ± 0.16
**A/G ratio (g/dL)**			
Control	1.63 ± 0.10	1.64^c^ ± 0.12	1.66^d^ ± 0.14
T1	1.63 ± 0.08	1.57^b^ ± 0.09	1.22^b^ ± 0.10
T2	1.56 ± 0.07	1.52^b^ ± 0.11	1.32^c^ ± 0.08
T3	1.56 ± 0.09	1.47^a^ ± 0.10	1.12^a^ ± 0.11
T4	1.54 ± 0.08	1.49^a^ ± 0.09	1.32^c^ ± 0.09
T5	1.59 ± 0.11	1.44^a^ ± 0.11	1.30^c^ ± 0.10
T6	1.61 ± 0.13	1.54^b^ ± 0.12	1.20^b^ ± 0.08

Chickens in the control group were fed the basal diet whereas the six treatment groups, in addition of basal diet received aqueous extract of *H. rhamnoides* in drinking water @ 100 mg/kg body weight of chicken (T1), @ 150 mg/kg body weight of chicken (T2), @ 200 mg/kg body weight of chicken (T3), @ 300 mg/kg body weight of chicken (T4), @ 400 mg/kg body weight of chicken (T5), and @ 800 mg/kg body weight of chicken (T6), respectively from days 0 to 42. Results are presented as mean ± S.E. Experimental unit 3 replicates pen (3 broiler chickens per replicate pen). Means bearing the different superscripts (^a, b, c, d^) in a columns differ significantly (*P* < 0.05).

Mean concentrations of plasma cholesterol and LDL were significantly reduced in treatment group birds as compared to control group at 21 and 42 days. Among the treatment groups, lowest concentration of cholesterol and LDL was recorded in plasma of T3 and T4 group birds (Table 6). Furthermore, significant higher level of HDL was recorded in treatment group birds and T3 group represents maximum HDL concentration. Moreover, no differences were observed in plasma triglyceride level among the experimental groups.

**Table 6:** Effect of *H. rhamnoides* extract on cholesterol, triglyceride, HDL and LDL level in broiler chickens.

Groups	0 day	21^st^ day	42^nd^ day
**Cholesterol (mg/dL)**			
Control	178.25 ± 05.23	169.67^c^ ± 04.92	165.32^d^ ± 03.88
T1	177.50 ± 08.42	158.00^b^ ± 04.30	138.50^b^ ± 02.59
T2	179.50 ± 10.96	155.17^b^ ± 04.78	139.67^b^ ± 02.19
T3	177.50 ± 13.24	142.50^a^ ± 04.98	128.50^a^ ± 03.30
T4	180.00 ± 07.56	147.57^a^ ± 06.16	131.67^a^ ± 02.24
T5	175.25 ± 07.92	158.67^b^ ± 06.47	137.00^b^ ± 02.64
T6	178.00 ± 07.39	158.00^b^ ± 05.81	143.67^c^ ± 02.71
**Triglyceride (mg/dL)**			
Control	138.87 ± 05.49	130.25 ± 07.15	123.25 ± 06.96
T1	136.63 ± 06.16	127.38 ± 05.68	120.50 ± 05.48
T2	131.93 ± 04.94	123.97 ± 05.05	120.00 ± 05.96
T3	135.21 ± 06.83	128.17 ± 09.83	121.00 ± 08.22
T4	132.48 ± 05.81	121.33 ± 08.96	118.75 ± 08.11
T5	134.87 ± 05.19	123.94 ± 07.11	119.25 ± 06.04
T6	135.92 ± 05.94	127.16 ± 06.37	123.00 ± 04.91
**HDL (mg/dL)**			
Control	19.81 ± 0.80	20.06^a^ ± 0.77	20.40^a^ ± 0.84
T1	19.70 ± 0.73	27.16^b^ ± 0.80	31.64^b^ ± 1.04
T2	20.04 ± 0.69	26.19^b^ ± 0.75	31.56^b^ ± 0.82
T3	19.50 ± 0.86	30.56^c^ ± 0.85	44.17^d^ ± 1.02
T4	20.16 ± 0.71	27.49^b^ ± 0.81	38.12^c^ ± 0.90
T5	19.71 ± 0.81	26.83^b^ ± 0.85	37.89^c^ ± 0.91
T6	19.39 ± 0.71	27.07^b^ ± 0.80	32.23^b^ ± 0.85
**LDL (mg/dL)**			
Control	53.35 ± 1.07	51.86^c^ ± 0.96	50.43^c^ ± 0.88
T1	53.91 ± 0.90	44.80^b^ ± 1.01	41.05^b^ ± 0.92
T2	54.12 ± 1.15	46.04^b^ ± 1.21	40.59^b^ ± 1.10
T3	53.80 ± 1.01	42.70^a^ ± 0.89	36.24^a^ ± 0.95
T4	53.26 ± 0.91	42.91^a^ ± 1.03	37.12^a^ ± 1.07
T5	54.07 ± 1.10	45.77^b^ ± 1.04	40.25^b^ ± 0.90
T6	53.90 ± 0.97	45.90^b^ ± 0.90	41.18^b^ ± 0.83

Chickens in the control group were fed the basal diet whereas the six treatment groups, in addition of basal diet received aqueous extract of *H. rhamnoides* in drinking water @ 100 mg/kg body weight of chicken (T1), @ 150 mg/kg body weight of chicken (T2), @ 200 mg/kg body weight of chicken (T3), @ 300 mg/kg body weight of chicken (T4), @ 400 mg/kg body weight of chicken (T5), and @ 800 mg/kg body weight of chicken (T6), respectively from days 0 to 42. Results are presented as mean ± S.E. Experimental unit 3 replicates pen (3 broiler chickens per replicate pen). Means bearing the different superscripts (^a, b, c, d^) in a columns differ significantly (*P* < 0.05).

A significant lower glucose level was recorded in treatment group birds as compared to control group at 21 day (Table 7). No differences were observed in the mean values of creatinine and ALT among the experimental groups whereas, AST level was reduced in T3 and T4 group birds as compared to control group.

**Table 7:** Effect of *H. rhamnoides* extract on glucose, creatinine, AST and ALT level in broiler chickens.

Groups	0 day	21^st^ day	42^nd^ day
**Glucose (mg/dL)**			
Control	322.75 ± 12.45	311.75^d^ ± 9.85	309.50^d^ ± 6.11
T1	324.00 ± 13.63	291.25^c^ ± 6.30	280.25^c^ ± 4.28
T2	321.75 ± 13.02	274.00^b^ ± 9.05	260.00^b,c^ ± 9.46
T3	324.25 ± 14.41	249.25^a^ ± 6.79	213.00^a^ ± 3.87
T4	326.50 ± 12.41	266.25^b^ ± 6.32	253.00^b^ ± 4.43
T5	324.25 ± 13.77	294.25^c^ ± 7.16	300.75^d^ ± 6.23
T6	327.00 ± 12.94	297.25^c^ ± 7.25	302.25^d^ ± 9.91
**Creatinine (mg/dL)**			
Control	0.20 ± 0.05	0.30 ± 0.04	0.26 ± 0.03
T1	0.20 ± 0.04	0.27 ± 0.03	0.25 ± 0.02
T2	0.20 ± 0.06	0.30 ± 0.04	0.27 ± 0.02
T3	0.22 ± 0.05	0.28 ± 0.04	0.25 ± 0.04
T4	0.23 ± 0.03	0.31 ± 0.03	0.27 ± 0.03
T5	0.22 ± 0.02	0.27 ± 0.04	0.25 ± 0.05
T6	0.21 ± 0.02	0.31 ± 0.07	0.28 ± 0.05
**AST (IU/L)**			
Control	80.75 ± 7.58	60.00^b^ ± 2.79	75.00^b^ ± 4.67
T1	81.50 ± 6.39	62.25^b^ ± 3.70	74.00^b^ ± 6.48
T2	79.25 ± 4.64	53.25^a,b^ ± 7.71	67.00^a,b^ ± 5.47
T3	79.50 ± 7.96	45.25^a^ ± 4.19	54.75^a^ ± 4.05
T4	80.00 ± 7.49	45.25^a^ ± 4.62	56.75^a^ ± 5.32
T5	79.00 ± 6.27	58.00^b^ ± 3.81	61.25^a^ ± 4.11
T6	79.75 ± 6.81	57.25^a,b^ ± 3.25	63.75^a,b^ ± 4.93
**ALT (IU/L)**			
Control	18.25 ± 1.25	12.00 ± 0.40	12.25 ± 0.62
T1	18.75 ± 1.65	11.75 ± 1.03	11.00 ± 1.08
T2	18.50 ± 0.64	13.25 ± 0.62	10.75 ± 0.62
T3	17.75 ± 2.65	11.25 ± 0.62	09.75 ± 0.85
T4	18.00 ± 1.35	12.75 ± 1.65	11.00 ± 0.81
T5	17.75 ± 0.75	11.75 ± 0.85	10.25 ± 1.03
T6	18.25 ± 0.85	12.75 ± 1.03	10.00 ± 1.08

Chickens in the control group were fed the basal diet whereas the six treatment groups, in addition of basal diet received aqueous extract of *H. rhamnoides* in drinking water @ 100 mg/kg body weight of chicken (T1), @ 150 mg/kg body weight of chicken (T2), @ 200 mg/kg body weight of chicken (T3), @ 300 mg/kg body weight of chicken (T4), @ 400 mg/kg body weight of chicken (T5), and @ 800 mg/kg body weight of chicken (T6), respectively from days 0 to 42. Results are presented as mean ± S.E. Experimental unit 3 replicates pen (3 broiler chickens per replicate pen). Means bearing the different superscripts (^a, b, c, d^) in a columns differ significantly (*P* < 0.05).

## Plasma antioxidant status in chickens

### TAC

We observed a significant increase in TAC in treatment groups that were supplemented with *H. rhamnoides* extract compared with the control group at both 21 and 42 day (Table 8). Birds in the T3 group represent the maximum TAC throughout the experiment.

### DPPH scavenging activity

DPPH scavenging activity in treatment group birds was increased as compared to the birds in the control group at both 21 and 42 day. Within the treatment groups, birds in the T3 group represent the maximum scavenging activity (Table 8).

**Table 8:** Effect of *H. rhamnoides* extract on MDA, TAC, and DPPH free radical-scavenging activity in broiler chickens.

Groups	0 day	21^st^ day	42^nd^ day
**MDA (nmol/mL)**			
Control	8.61 ± 0.58	8.31^c^ ± 0.30	8.06^c^ ± 0.18
T1	8.58 ± 0.68	6.05^a^ ± 0.26	5.81^b^ ± 0.12
T2	8.59 ± 0.64	6.32^a^ ± 0.24	5.67^b^ ± 0.14
T3	8.61 ± 0.67	5.91^a^ ± 0.13	4.04^a^ ± 0.32
T4	8.63 ± 0.63	6.47^a,b^ ± 0.16	5.49^b^ ± 0.18
T5	8.59 ± 0.70	6.94^b^ ± 0.14	5.47^b^ ± 0.13
T6	8.97 ± 0.48	6.93^b^ ± 0.16	5.50^b^ ± 0.08
**TAC (µmol/L)**			
Control	1122.43 ± 12.11	1139.66^a^ ± 17.60	1186.32^a^ ± 17.35
T1	1120.59 ± 10.15	1328.63^d^ ± 17.18	1547.37^b^ ± 18.34
T2	1121.31 ± 07.20	1339.49^d^ ± 18.08	1665.58^c^ ± 18.80
T3	1119.32 ± 07.47	1414.76^e^ ± 20.30	1698.53^d^ ± 20.99
T4	1118.33 ± 08.89	1254.24^c^ ± 15.79	1613.26^c^ ± 20.71
T5	1123.83 ± 06.35	1266.81^c^ ± 16.50	1649.61^c^ ± 20.29
T6	1119.82 ± 05.62	1213.81^b^ ± 18.58	1605.79^b^ ± 20.25
**DPPH radical-scavenging activity (%)**			
Control	41.24 ± 0.83	42.17^a^ ± 2.01	44.88^a^ ± 1.99
T1	41.77 ± 0.80	51.59^b^ ± 1.61	62.83^c^ ± 3.25
T2	41.11 ± 0.57	52.29^b^ ± 0.96	62.17^b,c^ ± 0.90
T3	41.60 ± 0.98	57.44^c^ ± 0.80	65.37^c^ ± 1.74
T4	42.02 ± 0.74	50.24^b^ ± 0.87	60.55^b,c^ ± 2.39
T5	41.29 ± 1.03	51.38^b^ ± 0.81	58.91^b^ ± 2.46
T6	41.34 ± 0.69	49.07^b^ ± 2.29	55.78^b^ ± 0.80

Chickens in the control group were fed the basal diet whereas the six treatment groups, in addition of basal diet received aqueous extract of *H. rhamnoides* in drinking water @ 100 mg/kg body weight of chicken (T1), @ 150 mg/kg body weight of chicken (T2), @ 200 mg/kg body weight of chicken (T3), @ 300 mg/kg body weight of chicken (T4), @ 400 mg/kg body weight of chicken (T5), and @ 800 mg/kg body weight of chicken (T6), respectively from days 0 to 42. Results are presented as mean ± S.E. Experimental unit 3 replicates pen (3 broiler chickens per replicate pen). Means bearing the different superscripts (^a, b, c, d^) in a columns differ significantly (*P* < 0.05).

### LPO

The concentration of MDA was decreased in treatment groups as compared with control group and lowest MDA concentration was observed in T3 group birds at 42 day of age (Table 8).

## Discussion

*H. rhamnoides* plant is habituated to cultivate in stressful surroundings of high altitude and is tolerated to abiotic stresses and such stressful conditions could upregulate the pathway of synthesis of secondary metabolites which results increase in the antioxidant content of this plant^30^. In this study, DPPH and ABTS radical scavenging capacity of the *H. rhamnoides* extract was increased in a dose dependent manner, similar to positive control ascorbic acid. DPPH and ABTS^+^ are stable free radical and are widely used to measure antioxidant capacity of plant extracts. The DPPH assay is very simple and sensitive and has been widely used to test the ability of compounds as free-radical scavengers or hydrogen donors. The assay is based on the reduction of purple DPPH radical to 1,1-diphenyl-2-picryl hydrazine in the presence of hydrogen donating antioxidant^31^. Whereas, ABTS assay is based on the generation of a blue/green ABTS^+^ by oxidation of ABTS with potassium persulfate that can be reduced by hydrogen donating antioxidants^23^. *H. rhamnoides* extract was also found to be rich in total antioxidant capacity, which might be due to the occurrence of diverse range of phytomolecules. It has been highly described that polyphenolic content in plant herbs are associated with their antioxidant capacity^32^ and in this study, *H. rhamnoides* extract was found rich in total phenolics, flavonoids and carotenoids content. These results confirmed the previous reports that polyphenolic compounds are the prime phytomolecules in *H. rhamnoides* and due to these constituents, *H. rhamnoides* exhibit its pharmacological antioxidant properties^11^ which are important for the scavenging of free radicals under stressful conditions of high altitude.

The capability of a plant extract to activate lymphocyte proliferation and enhance cytoprotection against free radicals is mostly ascribed to its higher polyphenolic and carotenoids content^3,14^. Since the phenolics, flavonoids and carotenoids content was recorded in a higher amount in our extract and might be due to their synergistic effect these phytomolecules could have been stimulated the proliferation of T lymphocytes and boost the immune system. It also indicated the mitogenic activity of *H. rhamnoides* in chicken lymphocytic cells. Our results are in agreement with the reports of Geetha *et al*.^14^ and Dorhoi *et al*.^33^ where the *H. rhamnoides* extract modulated the lymphocytes proliferation in laboratory animals, respectively.

Adverse effects of high altitude hypoxia reduce the growth performance in broiler chickens^34^ and our present findings of low body weight in broilers also support this hypothesis. Our results are in agreement with the reports of our previous work from our laboratory where we found similar reduction in the body weight of RIR cross-bred broilers at high altitude^2,3,35^. The reduction in the growth performance might be because of altitude of the experimental site which lies under high altitude and coupled with low PO_2_. Moreover, the decrease in the body weight might be due to the reduction in energy intake and increase in energy expenditure at high altitude. This misbalance in energy utilization leads to decrease in body mass through poor intestinal malabsorption and increase in the catabolism which ultimately reduces the overall growth^2,3,35^.

Moreover, improvement in the birds that were supplemented with *H. rhamnoides* extract could have been due to the synergistic effect of phenolics, flavonoids, and carotenoids present in *H. rhamnoides* fruit extract which could help in the higher utilization of feed by stimulating increased digestion of nutrients in gastrointestinal tract of chickens^36^. Also due to its higher antioxidant property, *H. rhamnoides* may eliminate the production of free radicals which ultimately reduces the oxidative stress in poultry chickens and improves their growth performance^13^. The net economic return also disclosed higher profit in the treatment groups chickens with lesser mortality from ascites and coccidiosis. Authors strongly believe that supplementation of *H. rhamnoides* extract enhanced the anabolic rate and reduced the catabolic activities in broilers.

In order to determine the bird’s health status and their physiological responses to different stress conditions, analysis of their blood biochemical constituents are important indicators. The increased concentration of total protein in treatment group chickens could have been due to more nutrional content of *H. rhamnoides* fruit which causes higher protein synthesis. Albumin protein is a useful marker of inflammation^37^ and elevated level of albumin in treatment group chickens indicates anti-inflammatory activity of *H. rhamnoides*^38^. Globulins are produced by the immune system cells and higher globulin concentration in treatment groups indicated the immunomodulatory property of *H. rhamnoides* extract under stressful conditions^14^. Ratio of A/G was decreased in treatment groups and this might be due to increase globulin concentration, which also indicates improved immunity in birds.

In the present study, *H. rhamnoides* extract reduced the level of plasma cholesterol and LDL whereas; increased the level of HDL in treatment group birds. HDL are referred as good cholesterol as it transport excess cholesterol from peripheral tissues to liver for its conversion in to bile acid whereas LDL are referred as bad cholesterol and its main function is to transport the cholesterol from liver to peripheral tissues^39^. Reduced level of LDL and enhanced level of HDL in treatment group birds might be due to the ability of polyphenolic compounds of *H. rhamnoides* to decrease and increase the secretion of apolipoprotein B (apoB) and apolipoprotein A-1(apoA-1), respectively^40^. The reduced level of cholesterol might be due to the inhibitory effect of *H. rhamnoides* flavonoids on the activity of 3-hydroxy-3-methyl-glutaryl-coenzyme A (HMG-CoA) reductase and acetyl coenzyme acetyl transferase, key regulatory enzymes in cholesterol biosynthesis^41^.

Similarly, *H. rhamnoides* extract decreased the glucose level in birds and this might be due to reduce gluconeogenesis with decrease in the glucocorticoid secretion by *H. rhamnoides* flavonoids^42^. Plasma creatinine level did not differ among the groups which exhibit non-toxic and non-pathological effect of *H. rhamnoides* extract on kidney. ALT and AST are intracellular enzymes accruing in liver and heart, and their enhanced activity is the indication of liver and heart damage^43^. In the present study, ALT level was remained unchanged whereas concentration of AST was decreased with supplementation of *H. rhamnoides* extract. This could have been due to hepatoprotective activity^13^ of *H. rhamnoides* extract in birds liver cells.

In this study, *H. rhamnoides* extract was supplemented to broiler birds as a source of antioxidant and the extracts was found to possess higher TAC and free radical scavenging capacity, as determined by reduced DPPH and ABTS activity. Additionally, antioxidant parameters such as MDA, FRAP, and DPPH were analysed too in blood plasma samples of chickens for determining the effect of *H. rhamnoides* extract on antioxidant enhancement. Results indicated that supplementation of *H. rhamnoides* in broilers enhanced the level of TAC and free radical scavenging activity while reduced the level of MDA in plasma samples. In free radical scavenging process the antioxidants present in *H. rhamnoides* may prevent oxidation of biological molecules by reducing the rate of free radical chain initiation e.g., either by scavenging initiating free radicals or by stabilizing transition metal radicals such as copper and iron^6^. Moreover, increased in the antioxidant defense level and deceased in the oxidative stress marker MDA might probably due to the synergistic effect of phenolics, flavonoids, and carotenoids that were present in *H. rhamnoides* fruit extract. Earlier reports of Purushothaman *et al*.^44^ and Zhou *et al*.^45^ revealed a marvellous depletion in MDA concentration and higher antioxidant defense level in animals with the supplementation of *H. rhamnoides* extract. *H. rhamnoides* extract provides protection from oxidative stress due to its higher antioxidative activity with its ability to scavenge free radicals^46^ and its ability to reduce the activation of caspase-3, downregulating the expression of pro-apoptotic genes Bax, and upregulating the expression of anti-apoptotic genes Bcl-2^47^. Better growth performance in broilers in this study could also be connected with potent antioxidative properties of the *H. rhamnoides* extracts. Hence, *H. rhamnoides* has beneficial effect on nutrient digestibility and scavenging of free radical and therefore, could be applicable as a broilers feed additive at high altitude.

In conclusion, this is a first report that demonstrated the feed additive potential of *H. rhamnoides* fruit extract in broiler chickens at high altitude. *H. rhamnoides* extract containing phenolics, flavonoids, carotenoids content ameliorate the hypobaric hypoxia induced reduced growth performance in broiler chickens through attenuating the effect of oxidative stress. *H. rhamnoides* extract improves the antioxidant defense level in broilers which also contributes to increase growth. Net economic return revealed the higher profit in *H. rhamnoides* supplemented groups because of lesser mortality. Moreover, *H. rhamnoides* extract at dose concentration of 200 mg/kg body weight of chicken has shown better effect as compared to other dose regime. Therefore, considering the beneficial effects of *H. rhamnoides*, a new feed additive may be prepared/formulated using the same extract in certain ration or preparation.
